# Influence of Plastic Machining on the Quality of a Clinching Joint in Aluminum

**DOI:** 10.3390/ma19040656

**Published:** 2026-02-09

**Authors:** Waldemar Matysiak, Jakub Kowalczyk

**Affiliations:** 1 Faculty of Mechanical Engineering, Institute of Materials Technology, Poznan University of Technology, 61-131 Poznan, Poland; 2 Faculty of Civil and Transport Engineering, Institute of Machines and Motor Vehicles, Poznan University of Technology, 61-131 Poznan, Poland; jakub.kowalczyk@put.poznan.pl

**Keywords:** metal forming, plate rolling, clinch, ultrasonic testing, ultrasonic testing

## Abstract

Permanent joints are commonly used in vehicle construction. The main methods used are gluing, welding and heat sealing. However, other joining methods that utilise plastic deformation of sheet metal are also becoming increasingly common. These methods include the sheet metal clinching joint. This is a joint that allows sheet metal to be joined, often in combination with bonding. Sheet metal of various thicknesses is used in vehicle construction and is subjected to plastic working. The main objective of the work was to assess the influence of plastic working of sheet metal on the strength of clinching joints. An additional objective was to determine the possibility of assessing the degree of aluminum sheet thickness reduction in a non-destructive manner using the ultrasonic (UT) method. The tests carried out showed that as the crumple increases, the strength of the clinched joint is reinforced. For sheet metal joints without crumpling, the strength was 608 N, and for 20% crumpling, the strength increased to 645 N, while for 47% crumpling, the strength increased to 671 N. For the largest crumpling of 60%, the strength was 712 N. In terms of non-destructive assessment of the degree of sheet thickness reduction, it was found that the best measure is the speed of the ultrasonic longitudinal wave. Other measures, such as decibel drops in pulse height for surface and longitudinal waves, show certain trends, but they are not conclusive.

## 1. Introduction

In the automotive industry, permanent joints of sheet metal components are most commonly produced by welding, resistance welding and various mechanical joining methods, including clinching. These joining technologies must provide sufficient strength, tightness and durability under complex loading conditions, often while being compatible with lightweight materials and high-volume production [1]. In particular, mechanically formed joints that rely on local plastic deformation have gained importance in lightweight body structures and multi-material systems [2,3].

Fusion welding relies on local melting of the base material, usually with the addition of a filler metal, followed by solidification to form a weld bead. Depending on the heat source and shielding method, numerous arc welding variants are used in body-in-white and structural applications. A welded joint consists of three main regions: the base material, the weld metal and the heat-affected zone (HAZ). While the base material does not undergo microstructural changes (for steels typically below about 500 °C), the weld metal is fully remelted and resolidified, and the HAZ experiences a complete thermal cycle without melting. As a consequence, the joint is heterogeneous in terms of microstructure and mechanical properties, which depend on the alloy, filler metal and welding parameters [4].

In alloys without allotropic transformations (e.g., aluminium, copper, austenitic and ferritic stainless steels), the HAZ is mainly characterised by grain growth and possible precipitation phenomena. In austenitic and ferritic steels, sensitisation due to chromium carbide precipitation or formation of intermetallic phases may lead to a loss of corrosion resistance and embrittlement. In plain carbon and low-alloy steels, where phase and allotropic transformations are present, the HAZ can be further subdivided into subzones of distinct microstructures and properties. Single-pass welds typically exhibit a cast, often dendritic microstructure, whereas multi-pass welds undergo self-tempering: previously deposited layers are reheated, resulting in partial normalisation of both the weld metal and HAZ. This can improve impact toughness and ductility compared with single-pass welds, while yield strength remains similar [4].

The main advantages of weld-based joining include relatively easy adjustment of process parameters, high efficiency in mass production, continuous visual control of the arc and weld pool, applicability to a wide range of materials and good suitability for automation and robotisation. However, welding is associated with several disadvantages, including environmental burdens. The process generates fumes and gases (e.g., NO_x_, CO_2_, metal oxides and other compounds), consumes significant amounts of energy—often from fossil sources—and produces solid waste such as spatter, electrode remains and flux residues. Improper waste management may lead to soil and water contamination, while noise and UV radiation can adversely affect both workers and nearby environments. Minimising these impacts requires modern process variants (e.g., laser or hybrid welding), adequate extraction and filtration systems, and responsible handling of consumables.

Mechanical resistance welding (e.g., spot or projection welding) is widely used to obtain permanent joints without the need for filler metal. In these processes, the parts are pressed together and locally heated by the passage of electric current, such that the material is brought to a plastic or near-molten state and joined under pressure. Resistance welding is characterised by low heat input, reduced influence of the atmosphere, relatively small changes in the base material microstructure compared with fusion welding, and high suitability for automation [4]. At the same time, it requires specialised equipment with relatively high investment costs, is best suited to certain material combinations (often similar metals), and is susceptible to defects when parameters are not properly controlled.

An alternative group of technologies for thin-sheet joining is the mechanical interlocking methods, among which clinching (stamped or press-fit joints) plays an important role. Clinching enables the joining of two or more sheet layers without additional elements such as rivets or adhesives. The process relies on local plastic deformation of the sheets using a punch and die set. The punch plastically deforms the material into the die cavity, forming a local bulge and undercut that mechanically locks the sheets together [1,2]. Variants of clinched joints include point, linear and combined (multi-point or multi-line) configurations, as well as dieless and roller-based solutions designed to tailor the deformation path [2,5,6,7].

Clinched joints offer several advantages: they do not require consumables, thus reducing material and process costs; they provide a relatively smooth and often visually unobtrusive connection; they minimise interruptions of protective coatings, which is beneficial for corrosion resistance; and they are suitable for high-volume, automated production. Thus, clinching is widely applied in the automotive sector, in the manufacture of white goods, housings and casings, and in various thin-sheet structures [1,2,3]. Recent studies have shown that the final neck thickness, interlock value and failure mode of clinched joints are highly sensitive to the local plastic strain distribution and deformation history of the sheets [3,8,9]. Numerical analyses and finite-element models of clinching confirm that small changes in the material flow path, friction conditions or local strength and ductility can markedly alter the joint geometry and load-bearing capacity [3,5,6].

Limitations include the requirement of relatively thin and ductile sheets (e.g., low-carbon steels and aluminium alloys), lower ultimate load-carrying capacity compared with some welded or riveted joints, and a limited applicability to very thick or very high-strength materials [2,7,8]. In particular, for aluminium alloys, both the intrinsic formability and any prior plastic deformation—resulting, for example, from forming or machining operations—affect the local flow behaviour during clinching and, consequently, the achievable neck thickness and interlock [2,5,10]. This provides the motivation for the present study, which focuses on the influence of plastic machining on the quality of a clinched joint in aluminium, by analysing how a controlled pre-deformation of the sheet modifies the local material state and, in turn, the geometry and mechanical performance of the joint.

One of the problems that has been decided to solve is how to assess whether the aluminum sheet used for clinching has been rolled. This is particularly important because rolling aluminum sheets significantly affects their mechanical properties [11,12]. It is not always possible in production conditions to perform metallographic tests or hardness measurements that would allow for an unambiguous determination of whether the sheet has been rolled [13].

Literature reports have shown that it is possible to assess the properties of sheet metal based on signals obtained during ultrasonic testing. Work has been carried out on the use of non-linear ultrasonic waves for non-destructive testing, including the use of surface waves. The authors [14] did not limit themselves to detecting discontinuities, but also assessed thermal stresses. The use of ultrasonic waves also includes the assessment of residual stress distribution in friction welding with mixing of aluminum plates [15]. In these studies, different ultrasonic wave frequencies (1, 2.5 and 5 MHz) were used. The work showed that it is possible to measure stresses, and importantly, that this can be performed using different frequencies. The authors [16] conducted a comprehensive analysis of the possibilities of testing partial stresses in materials. They introduced their own classification of methods. However, not all of the methods presented are suitable for use in testing aluminum sheets. For example, the method presented, which utilises the Barkhausen noise phenomenon, can only be used for ferromagnetic materials [17]. The work [18] not only confirmed the increase in the use of aluminum in modern industry, but also the possibility and need for ultrasonic testing. Research into changes in functional properties covers many different areas, from those related to health [19] to those related to agriculture [20], for strictly industrial applications [21,22].

The main objective of the work was to assess the impact of sheet metal forming on the strength of clinch joints. An additional objective was to determine the possibility of assessing the degree of aluminum sheet thickness reduction in a non-destructive manner.

## 2. Research

### 2.1. Research Procedure

**Figure 1:**
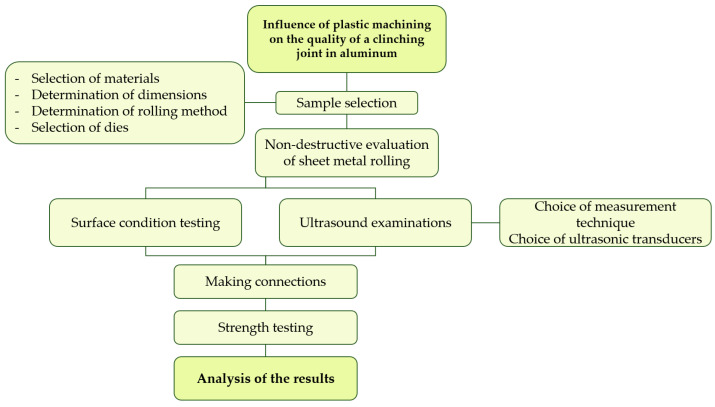
Research plan.

In order to achieve the set goal, research was planned and conducted. All work was carried out in accordance with the plan presented in Figure 1. First, the material for testing was selected, and samples were prepared. Next, their roughness and acoustic properties were tested using an ultrasonic method. In the next stage, the strength of the joints was prepared and then evaluated. This allowed conclusions to be drawn.

### 2.2. Materials and Methods

Aluminum strips were used in the tests. The sample dimensions were 120 mm × 20 mm. One crimped joint was assumed on each sample. Sheets with different crimping were used to make the samples. Four test series were planned with denting in the range of 0, 20, 47 and 60%. The test series designations, the initial sheet thickness before rolling and the thickness after rolling are presented in Table 1. Both sheets to be joined were of the same thickness.

**Figure 2:**
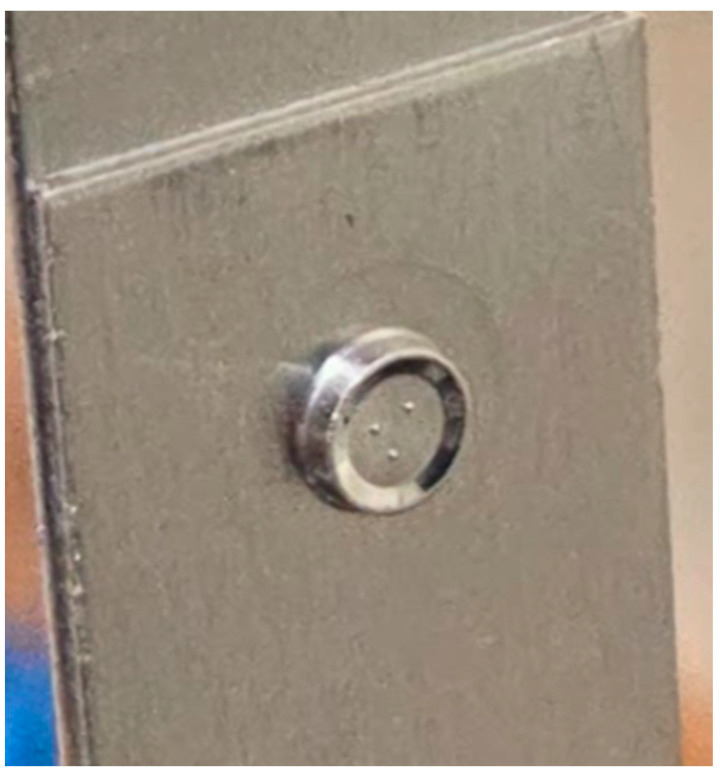
Example connection made for research purposes.

The samples were joined using cold pressing with a round punch and a suitably shaped die, resulting in a spot connection that requires no further processing and is free of burrs and sharp edges. The process is based on local plastic deformation of the material in the overlap area, without introducing thermal loads. The resulting joint does not cause degradation of the surfaces of the joined elements. The shaped joint is shown in Figure 2.

An aluminum sheet made of 6060 aluminium alloy was used in the preparation of samples. The chemical composition of the sheet is presented in Table 2. The tensile strength of 6060 sheet is above 130 MPa.

**Figure 3:**
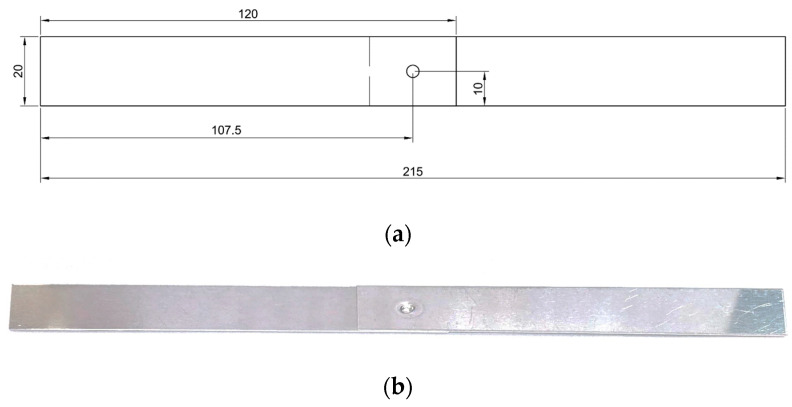
Samples used in the study: (**a**) sample model; (**b**) sample view.

**Figure 4:**
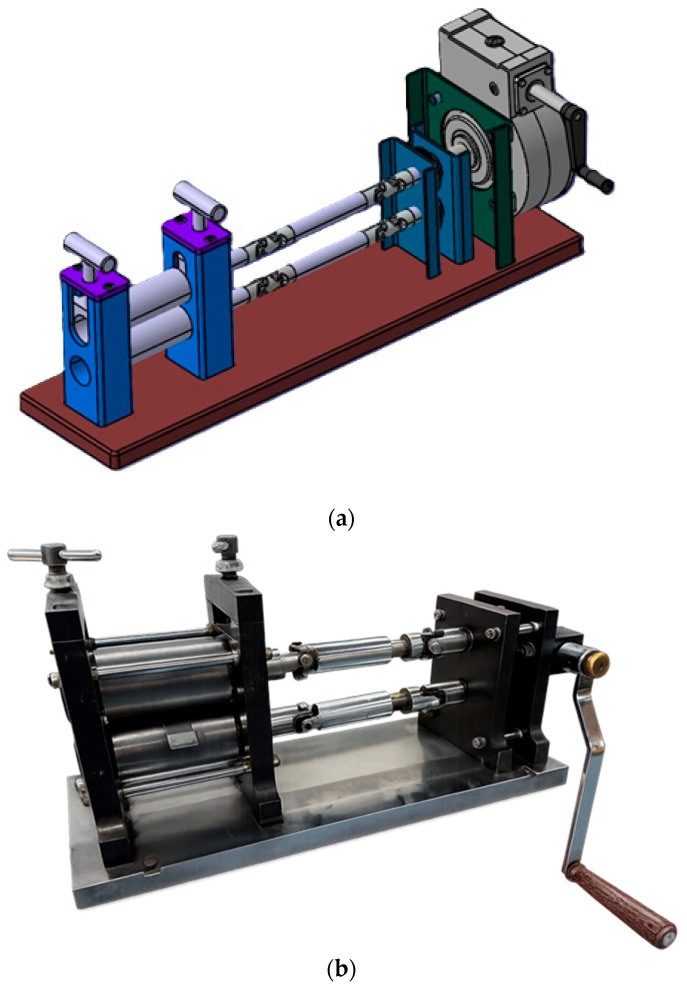
Rolling mill for rolling sheet metal 6060; (**a**) model; (**b**) view.

The metal strips were rolled and then cut to dimensions of 120 mm × 20 mm. After cutting, the metal strips were joined using a press-fit connection. A view of the finished sample is shown in Figure 3, and a view of the metal strip during rolling is shown in Figure 4.

Surface roughness measurements were performed to assess their impact on ultrasonic wave parameters. The average Ra roughness parameter of the sheet metal surface before crumpling was 0.17 µm, for 20% crumpling it increased to 0.19 µm and for 47% and 60% crumpling it was 0.23 µm and 0.46 µm, respectively.

The final thickness of all sheet metal strips was 0.8 mm. Four initial thicknesses of aluminium alloy sheet metal were used in the work, as follows: 0.8 mm for the first series (0% crumple), 1 mm for the second series (20% crumple), 1.5 mm for the third series (47% crumple) and 2 mm for the fourth series (60% crumple). Ten joints were made in each of the test series.

### 2.3. Ultrasound Examinations

After preparing the samples, the first step was to verify the possibility of testing the degree of aluminium sheet rolling in a non-destructive manner. Literature reports confirmed that it is possible to assess the condition of the material using the ultrasonic method. This method has been effectively used to measure both hardness and stress in materials [23,24]. Ultrasonic testing was also used to examine welded joints [25,26], which, like press-fit joints, generate concentrated stresses, and to examine coatings in car bodies [k20]. For the purposes of this study, it was decided that two ultrasonic techniques would be used. First, tests using ultrasonic surface waves were performed. The tests were carried out using digital flaw detectors—CUD (ZBM ULTRA sp. z o.o., Nadolice Małe, Poland) and USM35 (General Electric Company, Boston, MA, USA) and surface wave transducers with different frequencies (3.75 MHz pointed transducer and classic transducers: 4.22 and 8.02 MHz).

**Figure 5:**
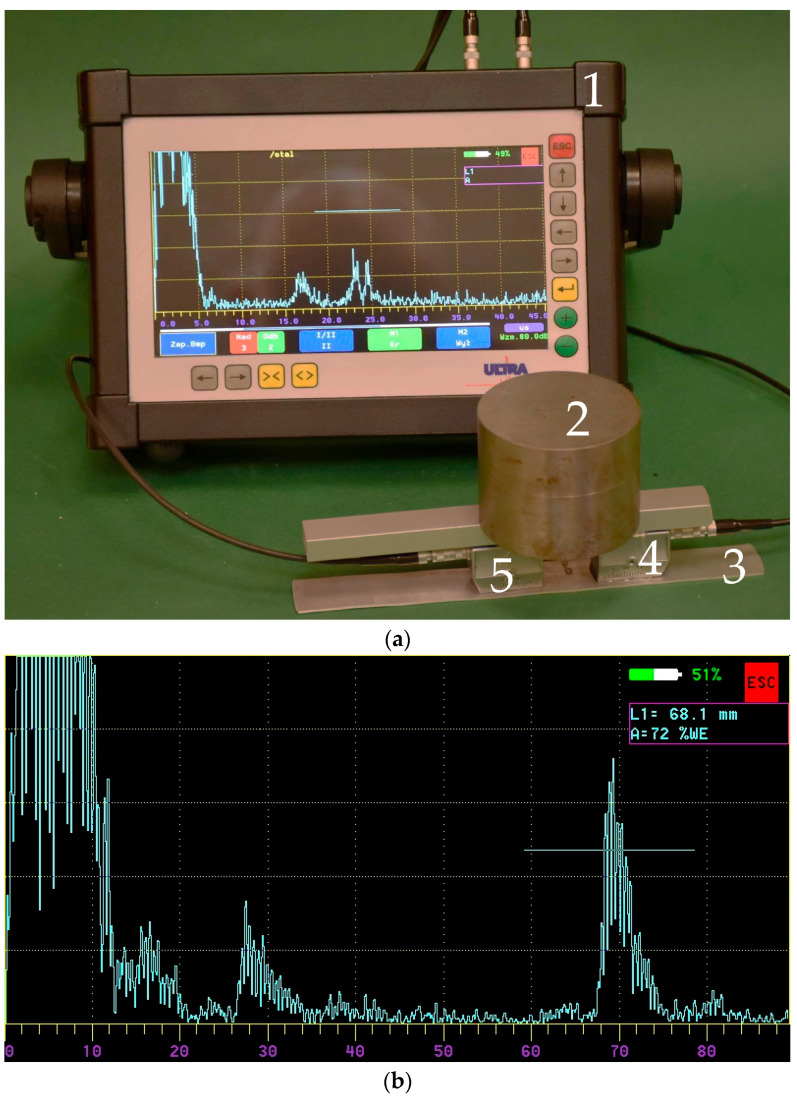
Test using classic surface wave transducers; (**a**) view of the test station 1—ultrasonic flaw detector, 2—load, 3—test sample, 4—transmitter head, 5—receiver head; (**b**) view of the screen with the obtained signal.

The work carried out using classic surface wave transducers confirmed that the ultrasonic wave transmitted from the transmitting transducer reaches the receiving transducer (Figure 5).

**Figure 6:**
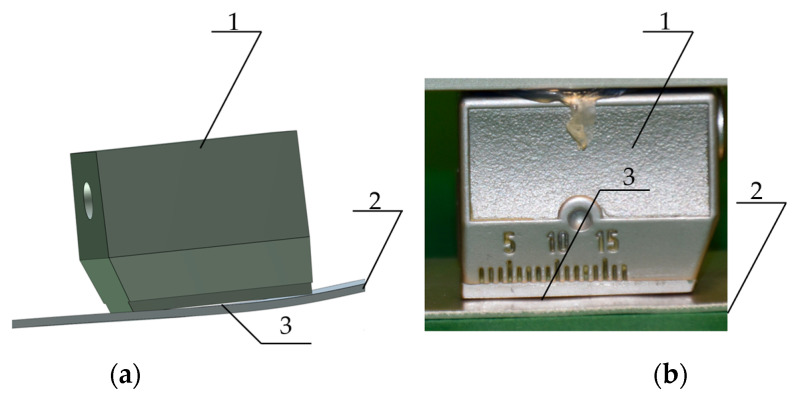
Location of the surface wave transducer; (**a**)—model; (**b**)—view 1—ultrasonic transducer, 2—aluminium sheet under test, 3—location where the transducer surface does not fully contact the surface of the sheet under test.

However, a significant error in measurements was observed, resulting from the slight deformation of the sheet metal (Figure 6). The lack of full contact and pressure across the entire surface of the probe means that the results obtained cannot be used in further work. Therefore, ultrasonic blade probes were used, which introduce the surface wave linearly.

**Figure 7:**
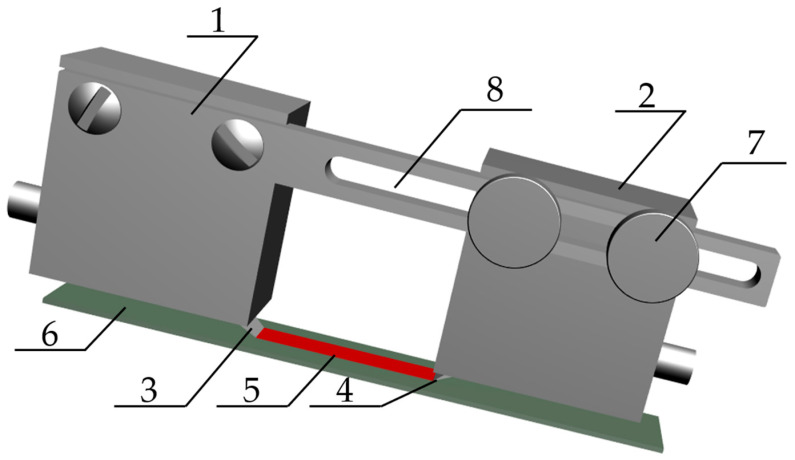
Measurement system model for a pointed probe: 1—transmitting probe, 2—receiving probe, 3—transmitter probe blade, 4—receiving probe blade, 5—Rayleigh wave, 6—test sample, 7—screws adjusting the distance between the probes, 8—guide.

Each time, a fresh coupling medium was applied to the edge of the probe blade after cleaning it. The probes were pressed with a constant force in all samples using a steel weight (weighing 500 g). The model of the measuring system is shown in Figure 7.

**Figure 8:**
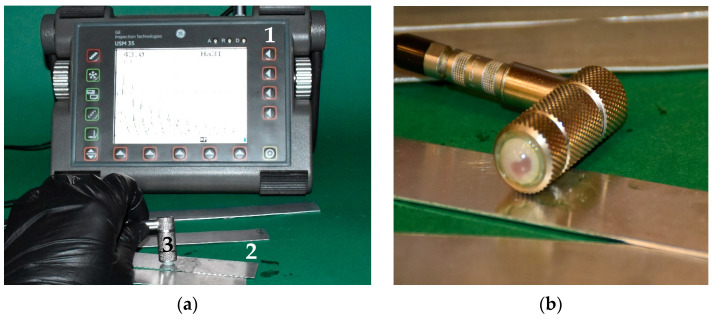
Measuring system: (**a**) view of the test station during testing, 1—ultrasonic flaw detector, 2—test sample, 3—ultrasonic probe; (**b**) ultrasonic transducer with water delay line.

In addition, ultrasonic echo technology was also used. Due to the small thickness of the samples tested, a high-frequency ultrasonic transducer with a water delay line was used. The flexible membrane on the transducer made it possible to obtain results regardless of the surface condition of the sample (Figure 8).

## 3. Results

### 3.1. Ultrasound Examinations

In the field of ultrasonic testing, it has been found that both the velocity of longitudinal ultrasonic waves and surface ultrasonic waves depend on the degree of rolling of the sheet metal. Increasing the degree of sheet rolling causes a significant decrease in the speed of the ultrasonic wave. A summary of these results is shown in Table 3, while the aggregate results are presented in Appendix A—Table A1.

At the same time, it was found that a similar downward trend in this velocity was observed for the surface wave. However, taking into account measurement errors, these changes are not as pronounced as for the longitudinal wave. The results for the surface wave are presented in Table 4.

**Figure 9:**
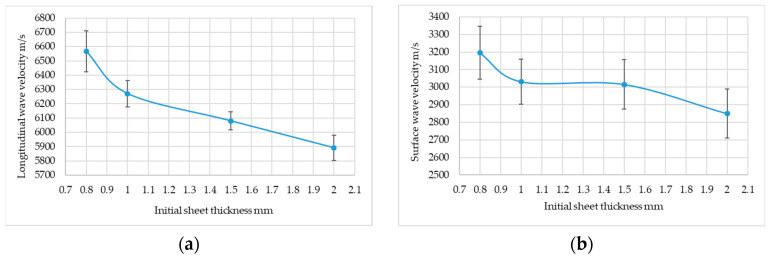
Comparison of the speeds of (**a**) longitudinal waves; (**b**) surface waves (visible confidence intervals).

Both trends can be clearly observed in the graphs shown in Figure 9.

In the area of longitudinal ultrasonic wave research, measurements of the height of the first four pulses on the ultrasonic flaw detector screen were also taken. These pulses were chosen because they are subject to a relatively small error. The decibel drop in height between successive pulses was taken as a measure, and the summary results are presented in Table 5.

**Figure 10:**
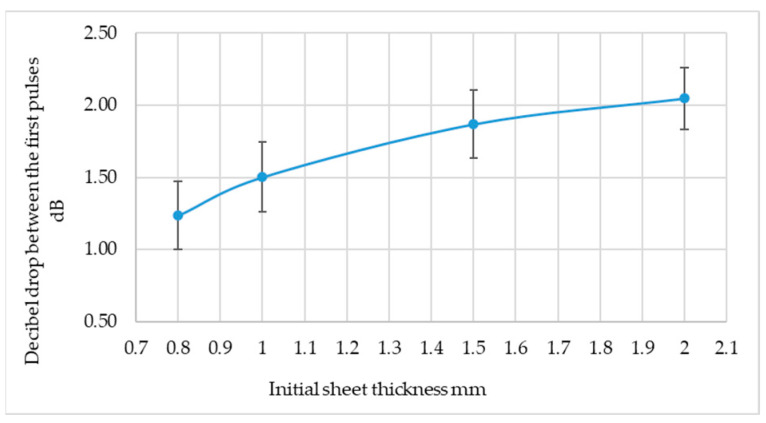
Change in decibel drop between the first two pulses on the flaw detector screen depending on the thickness of the sheet metal before rolling.

When analysing the results presented in Table 5, it can be seen that the smallest error was obtained for the decibel drop between the first and second pulses on the flaw detector screen (Figure 10).

**Figure 11:**
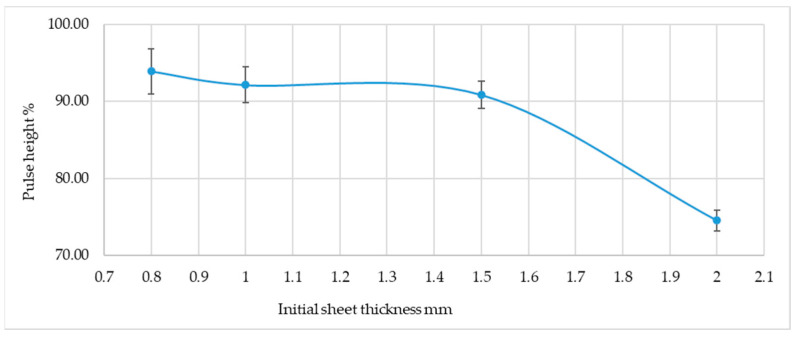
Change in pulse height on the flaw detector screen for a pointed probe depending on the thickness of the sheet metal before rolling.

The graph shown in Figure 11 confirms that there is an upward trend in attenuation with increasing sheet rolling. The results obtained are subject to considerable error. Definitely, the speed of the ultrasonic longitudinal wave is a better measure of sheet rolling than other measures.

### 3.2. Strength Testing

**Figure 12:**
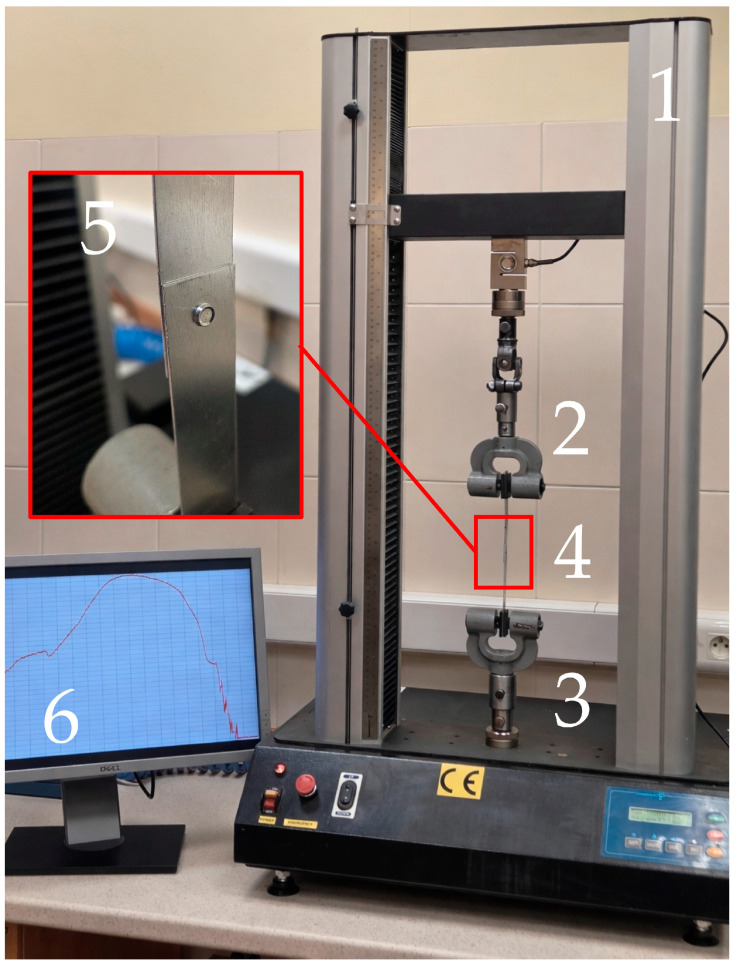
View of the testing machine during testing: 1—strength testing machine, 2—upper movable jaws, 3—lower fixed jaws, 4—sample, 5—connection view, 6—strength testing machine monitor.

The strength tests were carried out using a Cometech B1/E testing machine (Cometech Testing Machines Co., Taichung, Taiwan)—Figure 12. During the work, the breaking load of the tested joints was recorded. A total of 40 joints were tested, 10 joints from each group. For all tested joints, a similar relationship between force and displacement was obtained. The force was recorded after exceeding 50 N, which allowed for limiting the slack in the measuring system.

**Figure 13:**
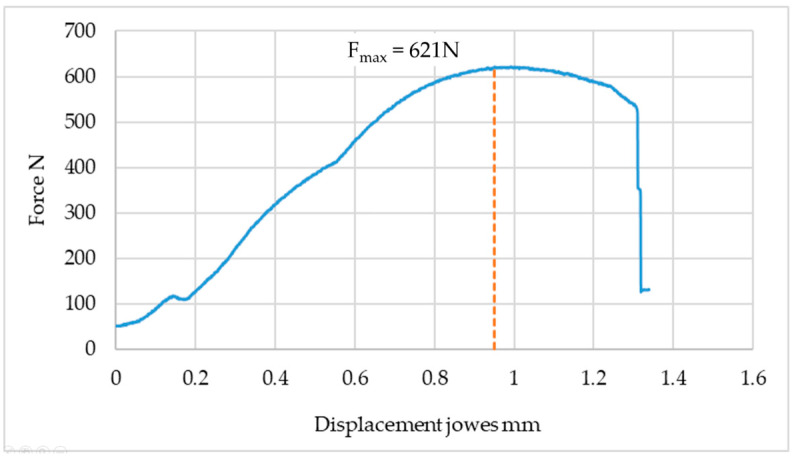
Example graph of force in the jaw displacement function of a testing machine.

An example graph of the breaking force as a function of displacement is shown in Figure 13.

**Figure 14:**
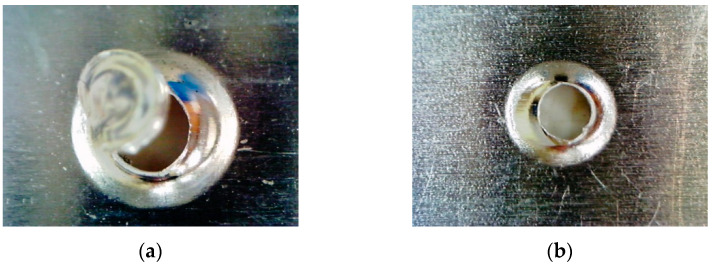
View of the joint after breaking: (**a**) first sheet, (**b**) second sheet.

The view of the connection after breaking is shown in Figure 14.

The results of the strength tests are presented in Table 6.

**Figure 15:**
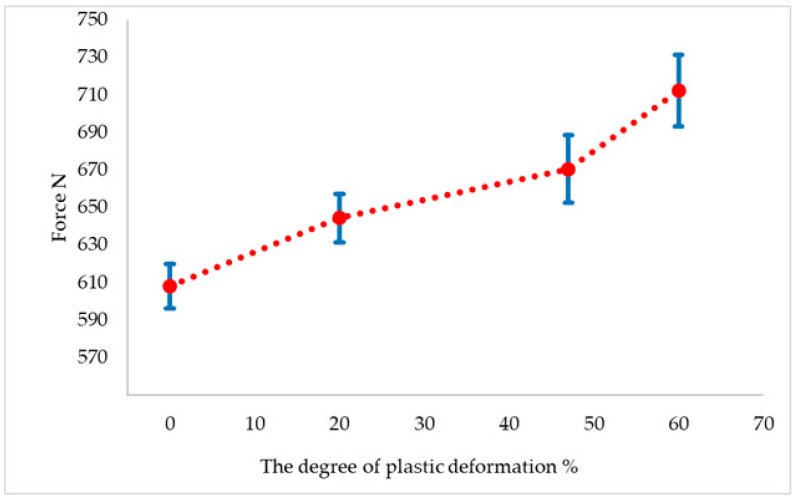
The average results of the strength tests.

The average results of the strength tests are shown in Figure 15.

Figure 13 shows that as the aluminum sheet is crumpled, the strength of the joint increases.

## 4. Analysis and Discussion of Results

The research covered two aspects. The first aspect involved examining the possibility of assessing aluminum sheet deformation using a non-destructive ultrasonic method. The work carried out showed that the denting of aluminum sheet metal causes a decrease in the speed of the ultrasonic wave, both for longitudinal and surface waves. It is worth noting that these changes are more significant for longitudinal waves. Research using surface waves has shown that the use of standard ultrasonic transducers is difficult due to the limited adhesion of the large surface of the transducer to the tested sheet metal.

An increase in roughness was observed, which is consistent with the literature [27,28,29]. Although a change in the Ra roughness parameter was observed in the range (0.17 to 0.46 µm), it does not affect the parameters of the signals obtained during ultrasonic testing [24].

The second aspect involved studying the effect of aluminum sheet crumpling on the mechanical strength of the joint. According to literature reports, it was noted that as the crumpling increases, the strength of the sheet increases. In [30], it was noted that after cold rolling, the grains of the AA 5052 alloy are elongated along the rolling direction. In addition, the authors showed that the tensile strength increases significantly and the elongation decreases markedly due to hardening during the cold rolling process. Similarly, in [31], it was noted that cold rolling increases the work hardening ratio and improves the mechanical strength and plasticity of the material. Also, in [32], it was noted that AA1050, which was heavily deformed by cold rolling at 50% and 66%, as well as under various metallurgical conditions, has significantly altered properties (including mechanical strength).

The increase in Ra roughness with increasing degree of crushing results from the intensification of plastic deformation of the sheet surface, leading to the development of irregularities associated with grain slippage and local material flow. With high crumpling, micro-irregularities and traces of contact with the rollers are additionally revealed and reinforced, resulting in a significant increase in the Ra parameter value.

At a displacement of 0.2 mm in each of the tested samples, a slight decrease in force of approximately 10 N was observed. This phenomenon is related to the mutual arrangement of the sheets in the connection area, which causes a temporary redistribution of stresses and a short-term reduction in the recorded force.

## 5. Conclusions

The paper presents the issue of plastic working of aluminum sheet metal (rolling) and its impact on the mechanical strength of crimped joints. The literature review and research conducted showed that:

As the crimp increases (in the range from 20 to 60 percent), the strength of the crimped joint increases. An increase in the force destroying the joint from 607 N to 712 N was observed.

The crumpling of aluminum sheet metal affects the change in surface roughness, but this change is minor and does not affect the functional properties of rolled sheet metal.

It was found that it is possible to assess the crumpling of aluminum sheet using a non-destructive ultrasonic method. Various ultrasonic measures of crumpling assessment were used in the ultrasonic tests. The high-frequency longitudinal wave velocity is the best way to assess the crumpling of aluminum sheet.

## Figures and Tables

**Table 1:** Designation of test series.

Designation	Initial Thickness mm	Final Thickness mm	Thickness Reduction %
Test series 1	0.8	0.8	0
Test series 2	1	0.8	20
Test series 3	1.5	0.8	47
Test series 3	2	0.8	60

**Table 2:** Chemical composition of metal sheet (%).

Designation	Fe	Si	Zn	Ti	Mg	Mn	Cu	Cr	Other	Al
6060	0.10–0.30	0.30–0.60	≤0.10	≤0.10	0.50	≤0.10	≤0.15	≤0.15	≤0.15	rest

**Table 3:** Results of longitudinal ultrasonic wave velocity measurements in the tested samples (the complete set of results is available in Appendix A, Table A1).

	Test Series 1	Test Series 2	Test Series 3	Test Series 4
Number of measurements	50	50	50	50
Minimum	6443	6195	6041	5822
Maximum	6758	6358	6117	5966
Average	6566.98	6271.34	6080.52	5892.10
Standard deviation	85.12	55.04	37.97	52.88
The t-student coefficient	1.68	1.68	1.68	1.68
Half confidence interval	142.71	92.27	63.66	88.65

**Table 4:** Results of surface velocity measurements of ultrasonic waves in the tested samples (the complete set of results is available in Appendix A, Table A2).

	Test Series 1	Test Series 2	Test Series 3	Test Series 4
Number of measurements	50	50	50	50
Minimum	3041	2864	2883	2707
Maximum	3358	3155	3167	2989
Average	3195.62	3031.66	3016.14	2849.88
Standard deviation	89.81	76.97	83.99	83.52
The t-student coefficient	1.68	1.68	1.68	1.68
Half confidence interval	150.57	129.04	140.81	140.02

**Table 5:** Ultrasonic measurements for the tested series (the complete set of results is available in Appendix A, Table A3).

	Test Series 1			Test Series 2			Test Series 3			Test Series 4		
	A_I_	A_II_	A_III_	A_I_	A_II_	A_III_	A_I_	A_II_	A_III_	A_I_	A_II_	A_III_
Number of measurements	50	50	50	50	50	50	50	50	50	50	50	50
Minimum dB	1.01	2.33	2.56	1.23	1.05	1.08	1.64	1.50	1.82	1.81	3.65	1.99
Maximum dB	1.52	3.29	4.05	1.72	2.03	2.41	2.18	2.50	3.36	2.36	4.57	3.96
Average dB	1.24	2.87	3.24	1.50	1.51	1.64	1.87	2.01	2.44	2.05	4.10	2.89
Standard deviation dB	0.14	0.24	0.37	0.14	0.23	0.29	0.14	0.25	0.33	0.13	0.27	0.50
The t-student coefficient	1.68	1.68	1.68	1.68	1.68	1.68	1.68	1.68	1.68	1.68	1.68	1.68
Half confidence interval dB	0.24	0.40	0.61	0.24	0.39	0.49	0.24	0.42	0.56	0.21	0.45	0.84

A_I_—decibel decrease in the height of the second pulse relative to the first, A_II_—decibel decrease in the height of the third pulse relative to the second, A_III_—decibel decrease in the height of the fourth pulse relative to the third.

**Table 6:** Results of strength tests for all test series.

	Test Series 1	Test Series 2	Test Series 3	Test Series 4
1	621 N	660 N	647 N	719 N
2	617 N	641 N	688 N	688 N
3	611 N	658 N	707 N	700 N
4	594 N	621 N	681 N	735 N
5	613 N	647 N	660 N	682 N
6	600 N	662 N	672 N	730 N
7	626 N	644 N	656 N	692 N
8	616 N	634 N	669 N	736 N
9	593 N	629 N	648 N	717 N
10	592 N	649 N	678 N	723 N

## Data Availability

The original contributions presented in this study are included in the article. Further inquiries can be directed to the corresponding author.

## Appendix A

**Table A1:** Summary of longitudinal ultrasonic wave velocity measurements in the tested samples for all test series.

Longitudinal Wave Velocity m/s			
Serious 1	Serious 2	Serious 3	Serious 4
6530	6276	6041	5893
6620	6276	6041	5893
6620	6358	6117	5893
6620	6276	6041	5822
6620	6276	6041	5893
6443	6195	6117	5966
6443	6276	6041	5893
6620	6276	6117	5893
6530	6358	6041	5893
6443	6195	6041	5822
6443	6195	6117	5966
6620	6195	6117	5822
6620	6276	6117	5822
6443	6358	6117	5893
6712	6276	6041	5822
6458	6195	6117	5893
6620	6276	6041	5966
6620	6358	6041	5966
6652	6276	6117	5966
6530	6276	6041	5822
6530	6276	6041	5822
6530	6276	6041	5966
6758	6276	6041	5966
6492	6195	6041	5893
6548	6276	6041	5893
6530	6195	6117	5893
6620	6276	6117	5893
6530	6195	6041	5893
6620	6276	6117	5893
6530	6358	6117	5966
6712	6276	6117	5822
6443	6195	6117	5893
6443	6276	6117	5822
6443	6276	6117	5966
6530	6358	6117	5822
6712	6276	6041	5966
6620	6358	6117	5893
6620	6358	6041	5893
6530	6195	6117	5893
6581	6358	6117	5893
6443	6358	6117	5822
6685	6276	6117	5966
6620	6276	6117	5893
6530	6276	6117	5893
6530	6195	6041	5822
6530	6276	6041	5966
6712	6276	6041	5822
6620	6195	6041	5966
6530	6276	6117	5822
6620	6195	6041	5893

**Table A2:** Summary of surface velocity measurements of ultrasonic waves in the tested samples for all test series.

Longitudinal Wave Velocity m/s			
Serious 1	Serious 2	Serious 3	Serious 4
3294	3136	3053	2738
3246	2928	2984	2977
3358	3020	3011	2799
3301	3066	2921	2822
3068	3081	2919	2764
3315	3037	3092	2775
3147	3035	2956	2707
3143	3154	3123	2763
3067	3085	3127	2730
3275	2987	2940	2922
3286	3155	2885	2825
3174	2949	3001	2893
3228	3147	3018	2889
3057	3068	3045	2761
3208	2974	3167	2707
3233	3046	3057	2861
3348	2999	3071	2892
3185	2882	2932	2928
3314	3032	3056	2732
3192	3146	3001	2789
3230	3018	3064	2931
3125	2979	3032	2904
3041	2945	2923	2933
3147	3089	2939	2950
3240	2989	3163	2898
3301	2864	2883	2710
3138	3035	3137	2948
3170	3092	2910	2905
3205	2977	3086	2955
3331	3142	3140	2989
3328	3081	3153	2909
3151	3041	3124	2819
3098	3095	2958	2728
3250	2933	2952	2908
3043	2927	3095	2888
3205	3087	2955	2832
3268	2924	3052	2843
3154	2931	3161	2828
3233	3120	3035	2914
3086	3112	3116	2906
3069	2995	2971	2817
3173	3126	3017	2803
3260	2957	2993	2876
3140	3105	3003	2951
3051	3027	2906	2989
3154	3133	3015	2763
3136	3022	2924	2889
3158	3009	2899	2944
3106	2941	2930	2769
3351	2960	2912	2721

**Table A3:** Summary of ultrasonic measurement results for the tested samples of all test series.

Serious 1			Serious 2			Serious 3			Serious 4		
A_I_	A_II_	A_III_	A_I_	A_II_	A_III_	A_I_	A_II_	A_III_	A_I_	A_II_	A_III_
1.1	3.3	3.6	1.5	1.6	1.1	1.8	2.1	2.8	2.0	4.6	2.6
1.1	2.8	3.1	1.4	1.9	1.2	2.1	1.9	2.1	1.9	4.2	3.4
1.1	2.7	3.9	1.5	1.3	1.7	1.9	2.0	2.2	1.9	4.6	3.2
1.3	2.6	3.3	1.5	1.4	1.6	2.2	1.5	3.1	1.9	4.5	2.2
1.4	2.9	2.6	1.3	1.8	1.3	1.6	2.2	2.1	2.4	3.9	2.3
1.4	2.8	3.1	1.4	1.6	1.9	1.7	2.2	2.1	2.1	3.8	3.0
1.4	3.0	3.3	1.3	1.5	2.2	1.8	1.9	2.1	2.3	4.2	2.0
1.4	3.0	2.7	1.5	1.3	2.0	1.9	2.2	2.3	2.0	4.0	2.8
1.4	3.0	3.3	1.6	1.5	1.7	1.7	2.4	2.4	2.1	3.9	2.8
1.3	2.5	3.9	1.6	1.1	1.4	1.8	1.9	2.6	2.1	3.7	4.0
1.4	2.5	3.7	1.7	1.5	1.6	1.6	2.5	2.0	2.1	4.2	2.4
1.2	3.1	2.7	1.7	1.3	2.0	1.7	2.4	2.4	1.9	4.2	2.8
1.1	3.0	3.2	1.3	1.8	1.1	1.8	1.9	2.5	2.1	4.2	2.9
1.1	2.9	3.4	1.6	1.7	1.6	2.1	2.0	2.6	2.0	3.8	3.8
1.0	3.0	2.8	1.4	1.9	1.5	2.1	1.6	2.6	2.2	4.0	2.6
1.2	2.6	3.3	1.6	1.3	1.5	1.9	1.8	2.6	2.1	4.4	2.1
1.3	2.6	3.2	1.5	1.4	1.7	1.9	2.1	1.9	2.2	3.7	3.1
1.5	2.8	3.2	1.7	1.2	1.9	2.0	1.6	2.4	2.3	3.9	3.0
1.1	3.0	3.9	1.7	1.1	2.4	2.0	1.7	2.9	2.2	3.7	2.6
1.1	3.0	3.5	1.6	1.5	1.6	2.0	2.0	2.4	1.9	4.6	2.3
1.4	2.4	3.6	1.6	1.3	1.6	1.8	1.9	3.0	1.9	4.3	2.6
1.3	2.7	3.6	1.4	1.7	1.5	1.9	1.9	2.4	2.0	3.7	3.5
1.1	2.9	3.0	1.6	1.5	1.3	1.8	2.4	2.4	2.1	3.9	3.5
1.4	2.6	2.8	1.7	1.3	1.8	1.6	2.3	2.3	1.8	4.2	2.9
1.4	2.5	3.8	1.5	1.5	1.4	1.8	1.9	3.1	2.2	3.8	3.3
1.2	2.9	2.7	1.3	2.0	1.7	2.0	1.7	3.0	1.9	4.5	2.7
1.2	3.2	3.0	1.2	2.0	1.3	1.9	2.0	2.3	2.2	4.1	2.7
1.2	3.0	3.0	1.2	1.9	1.8	1.7	2.1	2.5	2.1	3.8	3.2
1.0	2.7	3.3	1.4	1.7	1.8	1.9	2.0	2.0	1.9	3.8	3.9
1.0	3.0	2.9	1.6	1.7	1.2	1.7	2.2	2.7	2.0	4.2	2.8
1.0	2.9	4.0	1.5	1.4	1.6	1.9	2.0	1.8	2.1	4.2	2.3
1.3	2.8	2.7	1.7	1.4	1.9	1.9	2.2	2.1	1.9	4.3	3.2
1.4	2.3	3.2	1.4	1.5	1.5	1.8	2.1	2.3	2.0	4.6	2.4
1.1	3.1	3.4	1.6	1.6	1.6	1.9	1.6	3.4	2.1	4.0	2.2
1.0	3.2	3.5	1.4	1.8	1.2	1.9	2.0	2.2	2.0	4.2	2.9
1.4	2.9	3.4	1.4	1.5	1.8	1.9	2.0	2.6	2.1	4.0	2.4
1.4	2.8	3.4	1.3	1.5	2.0	2.1	1.5	2.1	2.1	4.3	3.5
1.2	3.2	2.7	1.4	1.8	1.6	1.8	2.2	2.3	1.9	4.3	2.3
1.4	2.9	3.1	1.6	1.3	1.8	1.8	1.8	2.6	2.1	4.4	2.9
1.0	2.9	3.5	1.7	1.3	2.2	1.9	1.8	2.7	2.3	3.8	2.8
1.3	2.9	3.1	1.4	1.6	1.5	1.9	1.9	2.4	1.8	4.1	3.1
1.4	2.5	3.0	1.6	1.4	1.4	1.8	2.3	2.2	2.1	3.7	3.6
1.0	3.1	3.5	1.5	1.3	2.1	1.8	2.4	2.2	2.1	4.4	2.4
1.2	3.2	2.8	1.5	1.3	1.6	1.9	2.1	2.2	2.0	3.9	2.6
1.2	2.6	3.8	1.7	1.2	1.6	1.8	2.4	2.6	2.0	3.8	3.3
1.2	3.2	2.9	1.6	1.4	1.8	1.7	2.3	2.5	1.9	3.9	3.8
1.2	2.9	3.2	1.3	1.6	1.4	2.1	1.8	2.8	2.0	4.4	2.6
1.3	3.2	2.9	1.4	1.7	1.3	1.7	2.1	2.2	2.0	4.1	3.3
1.1	2.7	3.0	1.4	1.5	1.7	2.0	1.8	2.7	2.1	4.1	2.9
1.4	3.0	3.3	1.7	1.3	1.9	2.0	1.7	2.4	2.0	3.9	2.8

A_I_—decibel decrease in the height of the second pulse relative to the first, A_II_—decibel decrease in the height of the third pulse relative to the second, A_III_—decibel decrease in the height of the fourth pulse relative to the third.
